# *CRELD1* variants are associated with bicuspid aortic valve in Turner syndrome

**DOI:** 10.1007/s00439-023-02538-0

**Published:** 2023-03-16

**Authors:** Catherina T. Pinnaro, Chloe B. Beck, Heather J. Major, Benjamin W. Darbro

**Affiliations:** 1 Stead Family Department of Pediatrics, University of Iowa, Iowa, IA 52242 USA; 2 Interdisciplinary Graduate Program in Genetics, University of Iowa, Iowa, IA 52242 USA

## Abstract

Turner syndrome (TS) is a chromosomal disorder caused by complete or partial loss of the second sex chromosome and exhibits phenotypic heterogeneity, even after accounting for mosaicism and karyotypic variation. Congenital heart defects (CHD) are found in up to 45 percent of girls with TS and span a phenotypic continuum of obstructive left-sided lesions, with bicuspid aortic valve (BAV) being the most common. Several recent studies have demonstrated a genome-wide impact of X chromosome haploinsufficiency, including global hypomethylation and altered RNA expression. The presence of such broad changes to the TS epigenome and transcriptome led others to hypothesize that X chromosome haploinsufficiency sensitizes the TS genome, and several studies have demonstrated that a second genetic hit can modify disease susceptibility in TS. The objective of this study was to determine whether genetic variants in known heart developmental pathways act synergistically in this setting to increase the risk for CHD, specifically BAV, in TS. We analyzed 208 whole exomes from girls and women with TS and performed gene-based variant enrichment analysis and rare-variant association testing to identify variants associated with BAV in TS. Notably, rare variants in *CRELD1* were significantly enriched in individuals with TS who had BAV compared to those with structurally normal hearts. CRELD1 is a protein that functions as a regulator of calcineurin/NFAT signaling, and rare variants in *CRELD1* have been associated with both syndromic and non-syndromic CHD. This observation supports the hypothesis that genetic modifiers outside the X chromosome that lie in known heart development pathways may influence CHD risk in TS.

## Introduction

Turner syndrome (TS) is a common, non-heritable genetic disorder affecting ~ 1 in 2000 females. TS is caused by a complete or partial absence of the second sex chromosome (Gravholt et al. 2022). Individuals with TS have one or more clinical manifestations, including short stature and ovarian insufficiency. Congenital heart disease (CHD) affects up to 45% of individuals with TS, with bicuspid aortic valve (BAV) encompassing about a third of CHD in TS (Fuchs et al. 2019). 45,X karyotype has the strongest association with CHD in TS (Fiot et al. 2019), however, this is neither necessary nor sufficient to cause CHD in TS. Documented mosaicism with a 46,XX cell line may mitigate CHD risk (El-Mansoury et al. 2007), but no definitive phenotype-karyotype correlations have been made with regards to CHD in TS (Gravholt et al. 2022).

There is evidence to suggest that X chromosome (Xchr)  haploinsufficiency sensitizes the genome and that TS-associated phenotypes may result from the interaction of Xchr and autosomal genes. Recently, it was shown that the presence of *TIMP3* variants (located on chromosome 22) are associated with BAV in TS– interestingly, individuals with TS who had both the deleterious *TIMP3* risk variant and who were haploinsufficient for *TIMP1*, an Xp paralog of *TIMP3*, had a 20-fold increased risk for BAV, which provides convincing evidence of sensitization. Additionally, genome-wide copy number variation is increased in TS, and a common, non-Xchr copy number variant (CNV) located on 12p13.31 encompassing *SLC2A3, SLC2A14,* and *NANOGP1* modifies the risk of left-sided CHD in TS (Prakash et al. 2016). Individuals with TS have global hypomethylation of their genome, with differential expression of autosomal genes suggesting that Xchr deficiency has genome-wide implications (Trolle et al. 2016). Furthermore, another recent study that compared differentially-methylated genes in individuals with TS and BAV, individuals with TS without BAV, and 46,XX individuals with non-syndromic BAV demonstrated significantly more differentially-methylated regions between individuals with TS with BAV and the 46,XX BAV individuals, which is consistent with a larger impact of Xchr monosomy on the epigenetic landscape (Gutierrez et al. 2022).

The goal of this study was to identify candidate genetic modifiers and pathways of CHD, specifically BAV, in TS. More specifically, we sought to discover non-Xchr loci at which DNA sequence variation influences the TS phenotype, which is typically associated only with haploinsufficiency for the Xchr (Riordan and Nadeau 2017). Similar studies have been successful in identifying genetic modifiers of CHD in trisomy 21, another population that seems to be sensitized to risk factors for CHD (Ackerman et al. 2012). TS has the highest prevalence of BAV among all syndromic BAV, making TS an ideal condition to explore the genetic modification of BAV (Niaz et al. 2018).

## Materials and methods

### Patients and phenotyping

Following approval by the University of Iowa Institutional Review Board, we employed TriNetX (https://www.trinetx.com), a global health research network, to query the electronic medical record for all living individuals with a diagnosis of TS who had ever received medical care at the University of Iowa. Interested individuals were screened for eligibility. Complete inclusion criteria were as follows: (1) confirmed diagnosis of TS by karyotype, (2) no known or suspected additional genetic diagnosis. The exclusion criteria were as follows: the presence of additional genetic abnormalities identified by karyotype, FISH, or chromosomal microarray, and (2) incomplete medical record (i.e., no previously performed transthoracic echocardiography). Informed consent was obtained from individuals 18 and older and parents of pediatric patients. Assent was also obtained from participants aged 7–12. Peripheral blood or saliva was then obtained for DNA extraction. A total of 28 eligible individuals were recruited. For the purpose of this study, we defined cases as individuals with TS who had BAV as determined by TTE clinically interpreted by a pediatric cardiologist per local protocol. Controls were individuals with TS without evidence of BAV or other CHD. 12 of 28 individuals were classified as cases.

Additionally, we obtained phenotypic data and corresponding genetic data from the “Whole exome sequencing to discover genetic variation associated with aortopathy in Turner syndrome–(study accession phs001531.v1.p1 in dbGAP). This TS cohort was assembled from the National Registry of Genetically Triggered Thoracic Aortic Aneurysms and Related Conditions (GenTAC) (Eagle 2009) and contained 188 individuals with TS. Eighty-eight were classified as having BAV by GenTAC, which employed a centralized imaging core and a single expert cardiologist to review cardiac imaging (Asch et al. 2016). Additional participant information regarding the TS subset of the GenTAC registry can be found here (Corbitt et al. 2018). 8 individuals from the GenTAC cohort were ultimately excluded from the analysis as their sequence read archive (SRA) data failed conversion to FASTQ files (2 cases, 6 controls). Thus, our final TS bicuspid aortic valve cohort was comprised of 208 individuals with TS (98 cases and 110 controls). See Table 1 for complete participant information.

**Table 1:** Demographic characteristics of Iowa and GenTAC TS cohorts

Cohort	% Cases (Bicuspid Aortic Valve Present)	% of overall cohort with 45,X karyotype
Iowa (*n* = 28)	42%	68%
GenTAC (*n* = 180)	47%	61%
All (*n* = 208)	47%	61%

28 individuals were recruited from the University of Iowa. Phenotyping and whole exome data for 188 individuals were obtained from dbGAP. 8 of 188 of those were not able to be included in the analysis given failed SRA to FASTQ file conversion (2 cases, 6 controls). A total of 208 individuals were included in the subsequent analysis. The majority of this combined cohort (61%) had karyotypes consistent with monosomy X (i.e., 45,X). Almost half of the combined cohort (47%) had bicuspid aortic valve, and the vast majority (> 95%) were Caucasian, non-Hispanic

### Whole exome sequencing

#### Iowa cohort

Genomic DNA was obtained either from lymphocytes isolated from whole blood samples or from saliva using standard laboratory methods. The samples were prepared for whole exome sequencing (WES) using the Illumina paired-end sample prep kit (Illumina, San Diego, CA) and captured using the Agilent SureSelect Human All Exon Version 5 Enrichment kit (Agilent Technologies, Santa Clara, CA). The Agilent kit captures 96.5 megabases. Samples were sequenced at the Iowa Institute of Human Genetics Genomics Division using an Illumina HiSeq 2500, generating 125-bp paired-end reads. Subsequent data analysis was performed using the Argon computer cluster at the University of Iowa. FASTQs were mapped to the GRCh38 human reference sequence (Broad bundle, accessed at https://console.cloud.google.com/storage/browser/genomics-public-data/resources/broad/hg38/v0) using Burrows-Wheeler Aligner (BWA-MEM, version 0.7.10). Local realignment and base quality score recalibration were performed using Genome Analysis Toolkit (GATK) (http://www.broadinstitute.org/gatk), and duplicate marking was performed using Picard tools (http:/picard.sourceforge.net). Haplotype caller was subsequently used to call genetic variants in standard Variant Call Format (vcf).

#### GenTAC cohort

Complete whole exome sequencing methods for the TS GenTAC cohort were described previously (Corbitt et al. 2018), but notably used the Roche Nimblegen SeqCap EZ to prepare the sequencing libraries, which captures 63.5 megabases. We downloaded SRA data through the dbGAP Authorized Access system, which was decrypted and converted to FASTQ files using the SRA toolkit (https://trace.ncbi.nlm.nih.gov/Traces/sra/sra.cgi?view=software). The resultant FASTQ files underwent identical processing as described above, with 8 samples failing conversion from SRA to FASTQ. To control for the differing exome capture libraries utilized in the two cohorts, we used bedtools (Quinlan and Hall 2010) to create an intersected bedfile containing only the overlapping areas of coverage between the two sequence capture libraries. We subsequently intersected all vcf files with the bedfile to ensure that called regions were present in both cohorts, which represented a coverage area of approximately 53 megabases.

Mapping statistics of the aligned reads and coverage of the shared exome target regions were subsequently analyzed using Qualimap software (García-Alcalde et al. 2012) (http://qualimap.bioinfo.cipf/es/). The average depth of Iowa samples was 103x, and the average depth of the GenTAC samples was 90x. We then used SAMtools (Li et al. 2009) (http://samtools.sourceforge.net) to calculate the mean coverage at each nucleotide and only included nucleotides with a coverage depth greater than 8 in both cohorts. Once the library- and depth-normalized vcf files were generated, they were subsequently filtered using GATK’s variant filtration function on quality by depth, mapping quality, and symmetric odds ratio. Variants passing the quality filters were run through Variant Annotation, Analysis and Search Tool (VAAST 2.0) (Hu et al. 2013), Sequence Kernel Association Test (SKAT) (Wu et al. 2011), and SKAT-O (Lee et al. 2012). We did not account for race-specific benign variants given that the vast majority of individuals included in our analysis were white, and non-Hispanic. We specified the target exomes as the cases (i.e., those with TS and bicuspid aortic valve) and the background exomes as the controls (i.e., those with TS but without bicuspid aortic valve).

### Candidate gene identification–VAAST 2.0

VAAST 2.0 uses an extended composite likelihood ratio test (CLRT) to determine a severity score for genomic variants, with the null model stating that the frequency of a variant is the same in target and background groups. The likelihood ratio is updated with conservation-controlled amino acid substitution frequencies, which results in the VAAST score (Hu et al. 2013). The p-value is then determined by permutation testing, as the CLRT becomes unnested with the incorporation of amino acid scoring data.

To ensure that there was no significant population stratification or issues with platform or variant calling procedures, we first measured the consistency of the background and target exomes. We then ran VAAST 2.0 in genome-wide mode with a maximum combined population for the disease-causing allele set at 0.05, dominant inheritance (which specifies that only the best scoring variant in each individual will be scored), and incomplete penetrance. Locus heterogeneity was permitted. The lowest hypothetical attainable *p*-value for a VAAST 2.0 run is governed by the number of unique permutations of exomes it can complete. This is governed by n choose k and equals 1/ n!/(k!/(n-k!), where n equals the total number of exomes and k equals the number of background exomes. With 208 total exomes and 110 background exomes, the minimum obtainable *p*-value approaches 1/infinity, indicating the study is adequately powered to achieve exome-wide significance.

The VAAST 2.0 output provides a list of candidate genes, ranked by VAAST 2.0 score and p-value. VAAST 2.0 p-values are corrected for linkage disequilibrium but do not account for multiple testing. As such, p-values were then corrected using the Benjamini–Hochberg method to control the false discovery rate (FDR) at 0.05. Statistically significant genes were then reviewed manually and analyzed with Ingenuity Pathway Analysis (IPA) software (QIAGEN Inc., https://digitalinsights.qiagen/com/IPA) (Kramer et al. 2014).

### Candidate gene identification–sequence kernel association tests

To achieve the most robust candidate gene identification, we also utilized the sequence kernel association test (SKAT) (Wu et al. 2011) and SKAT-O (Lee et al. 2012), the optimal unified test of SKAT. SKAT is a statistical model that tests for the association between a set of variants (rare and common) and continuous or binary phenotypes using a kernel machine method. The output is a list of genes ranked by *p*-value, which is calculated on a SNP-set (i.e., a gene or a region) by aggregating individual score tests on the SNP level while also adjusting for covariates. SKAT is particularly powerful when the genetic region has both protective and risk variants or many noncausal variants and with large sample sizes. SKAT-O was created because information on the directionality of variants is typically unknown and to maximize power in smaller exome-sequencing studies; it optimizes its performance based on the performance of the underlying burden tests and SKAT test. Prior to running SKAT, we used PLINK to create a genotype matrix and convert VCF to PLINK binary files (Purcell et al. 2007). To run these tests, we used R version 4.2.0 and implemented the SKAT package, using BAV as a binary trait and with default settings (R: A Language and environment for statistical computing. 2022).

As both SKAT tests and VAAST 2.0 provide ranked gene lists as output, we chose the top 2.5 percent of genes from each test (~ 50 genes) to evaluate for overlap. However, unlike VAAST 2.0, SKAT and SKAT-O do not consider contextually relevant variant characteristics, so we pre-filtered the variant list for the SKAT and SKAT-O analyses to only include only coding variants with a minor allele frequency < 0.05. Genes appearing in the top 50 in all three lists were manually reviewed and analyzed with the use of IPA (QIAGEN Inc., https:// digitalinsights. qiagen/ com/IPA) (Krämer et al. 2014).

## Results

### VAAST 2.0

The target and background datasets did not differ significantly with respect to the number of rare variants observed (*p* = 0.1, ratio of observed vs, expected 1.04), indicating that there were no systematic differences in the two groups.

The genome-wide run of VAAST 2.0 produced a list of 12 potentially significant candidate modifier genes that achieved exome-wide significance when using a Benjamini–Hochberg corrected *p*-value of 0.05 (Table 2).

**Table 2:** Statistically-significant genes from VAAST 2.0 output

Rank	Gene	VAAST score	FDR adjusted *p*-value
1	*CRELD1*	13.04	< 0.001
2	*ATG3*	13.02	< 0.001
3	*BPTF*	12.11	< 0.001
4	*ANKRD24*	12.72	0.02
5	*H1-1*	12.62	< 0.001
6	*MEX3D*	12.1	< 0.001
7	*P2RX4*	11.15	< 0.001
8	*ZFP41*	9.34	0.02
9	*PGM2L1*	9.23	< 0.001
10	*KBTMD12*	8.67	0.02
11	*C8orf34*	6.59	< 0.001
12	*ADAMTS18*	2.34	0.02

12 genes had an FDR-adjusted *p*-value < 0.05. Genes are ranked both by VAAST score and p-value. Higher VAAST scores suggest a higher likelihood of the gene containing disease-causing variants

These 12 statistically significant genes were manually reviewed by searching the gene name in Uniprot (Consortium TU 2021) for disease associations and in GTex portal (https://gtexportal.org/home/) for evidence of gene expression in the aorta or heart. We also searched all genes in Pubmed using the following Boolean search: “gene name” AND (“congenital heart disease” OR “bicuspid aortic valve” OR “heart development”). Additionally, all 12 genes were run through Ingenuity Pathway Analysis (Krämer et al. 2014) to look for known associations with heart development pathways and for involvement in the shared pathway(s). UniProt review of candidate genes demonstrated that *CRELD1* is associated with atrioventricular septal defects. No other genes had known disease associations identified in UniProt. GTex Portal review demonstrated that *CRELD1, ATG3, BPTF, P2RX4, ZFP41, PGM2L1,* and *C8orf34* all had some degree of aortic and/or cardiac gene expression. *CRELD1* literature search returned 12 results. All other searches returned 0 results. *CRELD1* was annotated in our pathway analysis as significantly related to cardiovascular system development (data not shown). *ADAMTS18* and *CRELD1* were both linked to heterotaxy/ciliopathy. *P2RX4* was listed as related to cardiovascular function. No other genes were identified to have a known association with CHD, BAV, or heart development.

### Intersection of VAAST 2.0, SKAT, and SKAT-O

**Fig. 1:**
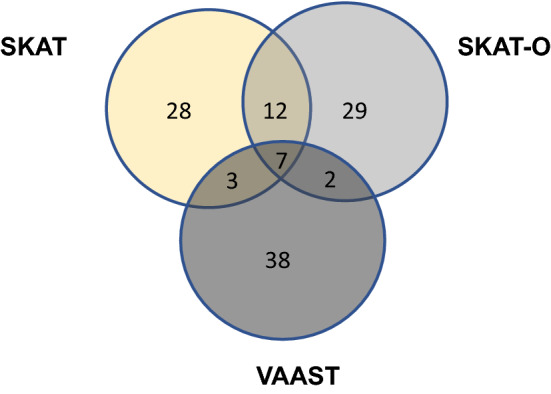
Overlap of gene burden tests for genetic modifiers of BAV in TS. There were 7 genes represented in the top 50 of all 3 gene burden analyses. 4 of these were statistically significant genes in the VAAST 2.0 analysis

Comparison of the three gene burden tests demonstrated 7 common genes among the top 50 genes: *CRELD1, DTNBP1, KBTBD12, P2RX4, PCDHB14, PGM2L1,* and *ZFP41.* Figure 1 illustrates the degree of overlap of genes across all tests.

*CRELD1*, *P2RX4, ZPF41*, and *KBTBD12* were previously reviewed as they were statistically significant in the VAAST 2.0 analysis. Manual searches using the above criteria of *DTNBP1, PCDHB14,* and *PGM2L1* retrieved no related articles. There was also no association with CHD, BAV, or heart development pathways noted in UniProt or from pathway analysis. *DTNBP1, PCDHB14,* and *PGM2L1* did all have some degree of aortic and/or cardiac expression when reviewed in GTex Portal.

Given that *CRELD1* achieved exome-wide significance in our VAAST analysis, was among the top 50 genes in all three gene burden tests performed, had published associations with CHD, and expression in the heart and/or aorta, we chose to evaluate the variants in this gene further. Three different rare variants in *CRELD1* were noted, which in total affected 14 cases and 2 controls and represented individuals from both cohorts. 1 individual was homozygous for the same *CRELD1* variant (Table 3).

**Table 3:** *CRELD1* (ENST00000682570.1, A0A804HKL1) DNA variants found in TS patients

Gene Name	Variants	# Individuals with variant	Frequency in gnomAD (v3.1.2)
*CRELD1* 3p25.3	c.9943412, G > A p.R309Q	9 cases, 1 individual is homozygous	0.01
	c.9943399, C > T p.Q305X	1 case	0.000539
	c.9944420, G > A p.D523N	4 cases 2 controls	0.00831

There are 16 individuals with rare variants *CRELD1* (14 cases, 2 controls)

## Discussion

Almost half of the patients with TS have CHD (Silberbach et al. 2018), demonstrating that TS is a significant risk factor for CHD. However, the existence of individuals with TS and structurally normal hearts demonstrates that an absent or structurally abnormal second Xchr is not sufficient to cause abnormal heart development. Increased, but variable penetrance may be a sign of interactions between the sensitized genome and modifier genes. Genetic modification of CHD is supported by observations in mice, as CHD incidence in mutant mouse models depends on their genetic background. For example, *Gata4*^±^ mice have a reported CHD incidence ranging from 12 to 76% (Rajagopal et al. 2007), *Nkx2-*^±^ between 5 and 50% (Winston et al. 2010), and *Tbx5*^±^ between 40 and 80% (Bruneau et al. 2001). In addition to incidence, CHD phenotype also varies with respect to mouse genetic background. For example, heterozygous *Gata4* mutations in mice can cause endocardial cushion defects, atrial or ventricular septal defects, hypoplastic right ventricle, or cardiomyopathy depending on genetic background (Rajagopal et al. 2007). Similarly, the phenotypic spectrum of *GATA4* mutations in humans is broad, indicating that genetic background (i.e., modifiers) may influence both the penetrance and expressivity of CHD risk genes in humans (Rajagopal et al. 2007).

Familial, non-syndromic BAV is phenotypically heterogenous and is inherited in an autosomal dominant pattern with incomplete penetrance (Huntington et al. 1997). Multiple genes/loci have been implicated in familial BAV, including *TGFBR2,* 5q15-21, *TGFBR1, NOTCH1, SMAD6, ACTA2,* 13q33-qter, 15q25-q26.1, *KCNJ2,* and 18q (Martin et al. 2007; Arrington et al. 2008; Gillis et al. 2017). Interestingly, Xchr loci are not strongly implicated in non-syndromic BAV despite the fact that TS has the highest prevalence of BAV among all genetic syndromes. This is also interesting given that non-syndromic BAV is more common in 46XY men (3:1) (Kong et al. 2017), which implies that absent Xchr genes may raise the risk for BAV but does not fully explain the phenotype. It also makes TS an ideal syndrome in which to look for genetic modifiers of BAV given the significantly enriched phenotype and thus increased power to detect rare modifiers.

In this study, we utilized VAAST 2.0, SKAT, and SKAT-O to identify rare autosomal candidate genetic modifiers of BAV in TS. We chose VAAST 2.0 for our bioinformatic analysis given its native integration of amino acid substitution, allele frequency, and phylogenetic conservation data to its variant association testing which improves power and variant prioritization accuracy (Hu et al. 2013). Because VAAST 2.0 was designed to be a general-purpose disease-gene finder capable of identifying both rare and common alleles responsible for both rare and common diseases, we chose to complement the VAAST 2.0 analysis with that of two alternative aggregative variant association tests (i.e., SKAT and SKAT-O).

*CRELD1,* which was the top-scoring gene in VAAST 2.0 and highly ranked in all 3 gene burden tests, has been implicated in both syndromic and non-syndromic CHD (Ackerman et al. 2012; Robinson et al. 2003; Zatyka et al. 2005), specifically atrioventricular septal defects. Creld1 controls the formation of the atrioventricular cushion and is required for the vascular endothelial growth factor (VEGF)-dependent proliferation of endocardial cells by promoting calcineurin-dependent nuclear translocation of nuclear factor of activated T cells 1 (NFATc1), and thereby, the expression of Nfatc1 target genes (Mass et al. 2014). *Nfatc1* null mice and mice lacking NF-ATc1 exclusively in endocardial cells fail to develop mature heart valves and is embryonic lethal. Conditional *Creld1* knockouts demonstrate that Creld1 controls the formation of the septa and valves but also the maturation and function of the myocardium at later developmental stages (Beckert et al. 2021). There is no association of *CRELD1, NF-ATc,* or calcineurin signaling with non-syndromic BAV. However, the aortic valve develops from the endocardial cushions in the outflow tract of the primitive heart tube (Martin et al. 2015). Since Nfatc1 has been implicated in the transcriptional regulation of valvulogenesis, it is plausible that on a distinct genetic background, variants in *CRELD1* could yield predominantly a BAV phenotype. Additionally, a set of 4 miRNA (miR-130a, miR-122, miR-486, and miR-718) have been correlated with BAV and aortic dilation (Abu-Halima et al. 2020) in TS and play a role in activating the TGF-β1 pathway and vascular remodeling mediated through vascular endothelial growth factor (VEGF-A) signaling pathway. *CRELD1* lies in the VEGF-A pathway, and rare variants in *CRELD1* and other genes in the VEGF-A pathway modify the risk of CHD in patients with trisomy 21 (Ackerman et al. 2012). This has been functionally validated in a mouse model of trisomy 21, whereby a null allele of *Creld1*, which has no heart or other phenotype on a disomic mouse model, greatly increases the occurrence of CHD in a mouse model of trisomy 21 (Li et al. 2012).

Interestingly, the duplication CNV associated with BAV in TS is also associated with conotruncal defects and left-sided lesions in 22q11.2 deletion syndrome suggesting that genetic modifiers of CHD may manifest differently on different high-risk backgrounds (Prakash et al. 2016). *CRELD1* has not been implicated in the pathogenesis of non-syndromic, non-familial BAV via GWAS (Helgadottir et al. 2018; Fulmer et al. 2019; Bjornsson et al. 2018; Yang et al. 2017; Gehlen et al. 2022; Gago-Díaz et al. 2017). Thus, the effect of *CRELD1* variants may be more penetrant in the setting of Xchr haploinsufficiency, representing an as-of-yet unidentified mechanism contributing to the polygenic heritability of BAV.

*CRELD1* is alternatively spliced. The 4 main transcripts expressed in the left ventricle and aorta are ENST00000452070.5, ENST00000397170.7, ENST00000435417.1, and ENST00000383811.7 (https://gtexportal.org/home/gene/CRELD1). The 3 variants in *CRELD1* enriched in our TS cases are interesting as the coding consequences for the variants are transcript-dependent. The first variant (c.9943412, G > A) would result in a synonymous proline-proline change in most *CRELD1* isoforms, however, in a specific isoform that codes for a shortened protein (337aa vs. 420aa) this change creates a nonsynonymous change of an arginine to a glutamine. The second variant (c.9943399, C > T) results in a nonsense mutation in both the canonical isoform and the cardiac isoforms. Many isoforms end prior to the third variant; in those transcripts it lies in the 3’ untranslated region. Interestingly, it results in missense variation (aspartate to an asparagine) in one isoform that is much larger than the canonical CRELD1 protein (537aa vs. 420aa). The significance of these isoform-specific consequences to BAV is not currently known and is the subject of further study.

Strengths of this study include a large sample size, and while 2 cohorts were assembled, significant quality measures were taken to minimize bias introduced by differences in study design. Weaknesses of this study include a lack of information regarding additional known CHD risk factors, including maternal age and maternal diabetes status. An additional weakness includes the potential to have misclassified BAV status in those we consider controls. Whereas misclassification would have decreased our study power and as such, our robust association of *CRELD1* is likely a valid finding. We opted to include all individuals with a karyotypically-confirmed diagnosis of TS given the lack of robust karyotype-phenotype correlation in TS, and the possibility of somatic mosaicism in those with only a 45,X cell line (Gravholt et al. 2022; Youssoufian and Pyeritz 2002).

Next steps include obtaining phenotypic data and DNA from biological parents and siblings of the Iowa cohort to determine whether the variants were inherited or occurred spontaneously and if there is an association with CHD in family members. Recurrence of damaging mutations would be consistent with the premise that genetic risk factors for CHD are present at low frequency in the general population. Unfortunately, it is difficult to recapitulate TS in mice given that a large portion of the human Xchr maps outside the mouse Xchr, the human pseudoautosomal region contains multiple genes that are autosomal in mice (Perry et al. 2001), and substantially more genes escape Xchr inactivation than in mice (15% versus roughly 3%) (Berletch et al. 2010). As BAV is also enriched in other genetic syndromes, such as Loeys-Dietz syndrome, validation could include a similar study investigating rare *CRELD1* variation in similarly sensitized BAV populations that have animal models (MacFarlane et al. 2019) available for later functional validation.

In conclusion, we propose that *CRELD1* variants show additive effects with haploinsufficiency for Xchr genes to interfere with valvulogenesis and increase the risk for BAV. The sensitized TS background aided the identification of the *CRELD1* modifiers, which have not previously been identified as associated with non-syndromic BAV. Further study of the VEGF-A pathway in syndromic and non-syndromic BAV may provide additional insight into the role of CRELD1 in the pathogenesis of BAV.

## Data Availability

The exome-sequence data with corresponding phenotype data generated during the current study are available from the corresponding author on reasonable request and are in the process of being transitioned to a database of genotypes and phenotypes (dbGAP). They will then be able to be requested directly with Institutional review board approval. The GenTAC exome sequence data and corresponding phenotype data were obtained from the database of genotypes and phenotypes (dbGAP) and can be requested directly from dbGAP with Institutional Review Board approval.
